# Occult Hepatitis B Infection: A Diagnosis to Have in Mind in Hemodialysis Patients

**DOI:** 10.7759/cureus.102083

**Published:** 2026-01-22

**Authors:** Andreia C Carnevale, Rita Birne, Ana Rita Martins, Jorge Dickson, Maria H Boquinhas

**Affiliations:** 1 Nephrology, Unidade Local de Saúde Lisboa Ocidental, Lisbon, PRT

**Keywords:** chronic kidney disease, ckd, hemodialysis, hepatitis b infection, occult disease, occult hbv

## Abstract

Hepatitis B infection (HBV) is a global health problem, with several distinct manifestations and risk of progression to cirrhosis or hepatocellular carcinoma. Patients with chronic kidney disease on hemodialysis are particularly vulnerable to infection due to the risk of disease transmission related to vascular access use, passage of blood through the extracorporeal circuit, shared dialysis equipment, and occasional transfusion of blood products. The clinical consequences, lack of therapy capable of complete eradication of HBV, and the fact that it is the viral disease most frequently transmitted via the parenteral route in this population make its timely diagnosis important. Frequent screening of HBV infection is recommended, and serological tests are usually sufficient for diagnosis of the disease. However, occult HBV infection is a clinical entity that can be difficult to diagnose. It is defined as the absence of detectable surface antigen (AgHBs), with positive HBV deoxyribonucleic acid (DNA) but with viral serum measurement <200 IU/mL, but this value can be much smaller, making its detection harder. It can also be categorized as seronegative if the core (AcHBc) and surface antigen (AcHBs) antibodies are both negative, or seropositive if one or both are positive. The latter can be important in the diagnosis, as it suggests previous exposure to the virus and should lead to measurement of HBV DNA, which might not occur with seronegative occult HBV infection. These characteristics may make the proper diagnosis of this disease difficult, with clinical and public health implications.

In this report, we present the case of an 85-year-old female patient with chronic kidney disease on hemodialysis. Routine screening of HBV AgHBs, AcHBc, and AcHBs had been previously negative. She was immunized with the HBV vaccine, and on the following screening, presented with negative AgHBs and AcHBc but positive AcHBs. After approximately two years of monitoring, an inconclusive measurement for AcHBc was detected. This prompted the assessment of HBV DNA, which was low but detectable (17 IU/mL). Reassessment, however, was negative (<10 IU/mL), with the other two measurements of 10 IU/mL (at the cutoff value, as defined by the laboratory) and later of 21 IU/mL. After these results, a diagnosis of occult HBV infection was considered.

This report is an example of the difficulty in diagnosing this entity, requiring a high index of suspicion that leads to HBV DNA measurement. Low circulating levels of HBV DNA can still result in inconclusive results, but other markers, such as a positive AgHBc, might aid us in establishing this diagnosis. Its early detection is important for proper differential diagnosis of reactivation or acute infection and later monitoring and management of the risk of reactivation due to persisting infection.

## Introduction

Hepatitis B infection (HBV) is a global health problem, with approximately two billion people who have been infected in the global population, of which 250 million live with the infection [1]. This disease has several distinct manifestations, from acute or fulminant hepatitis to chronic infection as an asymptomatic carrier, chronic hepatitis, cirrhosis, or progression to hepatocellular carcinoma [1]. Patients with chronic kidney disease on hemodialysis are particularly vulnerable to infection due to the risk of parenteral transmission, related to vascular access use, passage of blood through the extracorporeal circuit, shared dialysis equipment, and occasional transfusion of blood products [2]. Despite the implementation of preventive measures, such as vaccination, HBV infection remains a concern amongst hemodialysis patients due to the increased risk of transmission, clinical consequences, lack of therapy capable of complete eradication of HBV, and the fact that it is the viral disease most frequently transmitted via the parenteral route in this population [2,3]. In this context, frequent screening of HBV infection is recommended, with serological tests being usually sufficient for diagnosis and distinction between acute and chronic disease, previously cured infection, or post-vaccination status.

Occult HBV infection is associated with a particular serological profile that can make diagnosis difficult. It is defined as the absence of detectable surface antigen (AgHBs), positive or negative anti-core (AcHbc) or anti-HBs (AcHBs) antibodies, and positive HBV deoxyribonucleic acid (DNA) but with viral serum measurement <200 IU/mL [4-6]. However, HBV DNA value is much smaller (around 20 IU/mL) in about 90% of cases [7]. These characteristics may make the proper diagnosis of this disease difficult, requiring a high degree of suspicion that leads to HBV DNA measurement, which, however, may be too small to properly facilitate a definitive diagnosis. Other important differential diagnoses must also be excluded, such as reactivation of HBV or the initial stages of acute infection. With the prevalence of occult HBV infection in hemodialysis units estimated to be between 0.11% and 12.5%, with some studies suggesting up to 54%, and an associated risk of reactivation in certain circumstances, this matter becomes all the more relevant in this population [5,7,8].

This case aims to highlight the importance of detecting occult HBV infection in hemodialysis patients, the difficulties in reaching this diagnosis, requiring a high index of suspicion that leads to HBV DNA measurement, and the clinical implications.

## Case presentation

We report the case of an 85-year-old female patient with no previous relevant medical history other than chronic kidney disease on hemodialysis. As recommended, after initiation of dialysis, the patient was subjected to routine screening, including HBV, hepatitis C virus (HCV), and human immunodeficiency virus (HIV). AgHBs, AcHBc, and AcHBs were always negative, so she was immunized with four doses of anti-HBV vaccine in double dose, as recommended for patients in hemodialysis. On the following screening, she presented with negative AgHBs and AcHBc but positive AcHBs (with a maximum reported value of 58.67 IU/mL), suggesting an efficient immunization after the vaccine. HCV and HIV evaluations always remained negative.

After approximately two years without any clinical or analytical changes, an inconclusive measurement for AcHBc was detected. Despite the AgHBs remaining negative and AcHBs presenting with theoretically protective values, HBV DNA was assessed, with low but positive values (17 IU/mL). The diagnosis of occult HBV infection or acute infection was henceforward considered, and further study was requested. She was managed as infected and with risk of disease transmission, which required transferring her from her hemodialysis clinic to a hospital dialysis unit with a space and dialysis monitor device dedicated to HBV-positive patients. Reassessment was negative for HBV DNA (<10 IU/mL), so two other measurements were repeated, both of 10 IU/mL (at the cutoff value, as defined by the laboratory). Table 1 shows all the results pertaining to the HBV serological evaluation since initiation of dialysis (AgHBs, HBV e antigen [AgHBe], and AgHBe antibody [AcHBe] were always negative). Despite the inconclusive results, a final diagnosis of occult HBV infection was considered, further validated by another later measurement of HBV DNA of 21 IU/mL.

**Table 1:** HBV serological assessment Chronological evolution of the patient’s HBV serological assessment since initiation of hemodialysis. The laboratory cutoff values for AcHBs and HBV DNA were 5-12 IU/mL for inconclusive values and > 10 IU/mL for positive values, respectively.

Date	AcHBs (IU/mL)	AcHBc		HBV DNA (IU/mL)
		Total	IgM	
Day 1	inconclusive (10.55)	negative	–	–
Day 32	inconclusive (9.98)	negative	–	–
Day 205	positive (77.7)	negative	–	–
Day 390	positive (58.67)	negative	–	–
Day 755	positive (18.93)	inconclusive	negative	–
Day 807	Positive	–	–	17
Day 811	positive (29)	positive	negative	<10
Day 853	positive	–	–	–
Day 867	positive	–	–	10
Day 881	positive	–	–	10
Day 912	positive	–	–	–
Day 1137	–	–	–	21

A recent annual evaluation, which included abdominal echography, showed no relevant alterations, and further hepatic evaluation also showed no abnormal findings (with maximum Alanine Aminotransferase [ALT] and Aspartate Aminotransferase [AST] of 13 and 19 U/L, respectively). In this context, after consultation with Infectiology, it was considered that the risk of transmission was very low and the preventive measures regarding hemodialysis were no longer necessary. She was able to return to her previous dialysis clinic but was later transferred to another clinic, and no further follow-up information was available. 

## Discussion

This case report describes a patient on hemodialysis, vaccinated for HBV with serological assessment not suggestive of HBV infection (with no detectable AgHBs and negative AgHBe and AcHBe) and apparently effective immunization after vaccination (with increased AcHBs levels). Two years later, she presented with an inconclusive AcHBc value, despite the AgHBs remaining negative. Suspecting de novo or previously undiagnosed HBV infection, HBV DNA measurement was requested, which came back positive, suggesting a diagnosis of occult HBV infection. Its definition, proposed by the European Association for the Study of the Liver (EASL) in 2008, includes undetectable AgHBs, with or without positive AcHBc or AcHBs, and positive HBV DNA (identified via Polymerase Chain Reaction [PCR] technique, ideally from a sample of hepatic tissue, but serum DNA testing is considered sensitive) [4,8]. HBV DNA must be <200 IU/mL, with higher values being likely related to mutant HBV S variants that produce a modified AgHBs that is not recognized by commercially available tests and associated with overt HBV infection or reactivation [4,5,8]. Although this criterion was previously contested by some authors, the fact remains that in over 90% of cases, the measured serological values are closer to 20 IU/mL [7]. This characteristic of occult HBV infection, although rarely discussed, can impact proper diagnosis. In this reported case, the persistingly low values of HBV DNA, while consistent with what was previously discussed, are an example of the difficulty in detecting the infection.

The clinical implications of this diagnosis are not entirely clear. Most cases have low levels of HBV covalently closed circular DNA (cccDNA) in infected hepatocytes, with decreased viral replication and viral protein expression due to inhibition by the immune system and due to genomic regulation defects [1,7-9]. However, occult infection can still contribute to the development of cirrhosis and hepatocellular carcinoma, being prevalent in as much as 75% of patients with chronic liver disease and HBsAg-negative hepatocellular carcinoma and 4 to 38% of cirrhotic patients [6,8,9]. It is also associated with risk of reactivation during immunosuppressed states, in as much as 40% of cases when potent immunosuppressive therapies are used [5,8,9]. This can be relevant for hemodialysis in the context of possible future renal transplantation, where the need for immunosuppression (such as anti-CD20 therapy or calcineurin inhibitors) will require preventive measures [5,8]. Regarding the risk of transmission, it is not increased amongst hemodialysis patients and thus does not require isolation from other patients, as recommended by a statement from experts and the working group on viral diseases of the Spanish Society of Nephrology (SEN) [5]. However, an increasing HBV DNA level of >200 IU/mL and/or a positive AgHBs detected later will require isolation and referral for further evaluation [5]. While there is no evidence on the periodicity of monitoring, continued evaluation of the viral load is recommended, at least annually, but particularly in AcHBc-positive patients and those with a high risk of reactivation, such as those with high fragility, malnutrition, or immunosuppression [5,8].

This case also illustrates how other analytical alterations can lead to further study. HBV infection can be further categorized as seronegative or seropositive; while in the first case both AcHBc and AcHBs are negative (due to progressive loss of antibodies during infection or, theoretically, in cases that never developed them), in the second definition one or both antibodies are positive (from exposure to the virus, such as previous acute disease or chronic hepatitis with persisting HBV DNA) [4,8]. Seropositive HBV corresponds to 80% of cases and can help diagnose occult HBV infection, considering that both a positive AcHBc and occult HBV infection represent previous contact with the virus, while with seronegative cases, this association is harder to establish [1,7,8]. In this case report, that was the inciting event that raised suspicion and led to further study, despite the lack of signs or symptoms suggestive of hepatitis and the previous vaccination.

In other studies, the patients presented with similar characteristics, with most being seropositive (mainly for AcHBc), such as one showing a seroprevalence of AcHBc of 26.7%, of which 67.2% were AcHBs positive, further showing how a positive AgHBc is important for the detection of occult HBV, even when a positive AcHBs would indicate immunoprotection from HBV [10]. Some, however, report higher average HBV DNA loads, such as a study in Ghana that reported a mean viral load of 98.7±53.5 IU/mL, with the lowest value of 34 IU/mL, which would facilitate the diagnosis of occult HBV infection [11].

## Conclusions

This case report represents an example of the difficulty in a proper diagnosis of occult HBV infection. The undetectable AgHBs and positive HBV DNA with commonly extremely low values make these results hard to detect or interpret, but other markers, such as a positive AgHBc, might aid us in establishing this diagnosis. Its early detection, however, is important, particularly in vulnerable populations such as those of patients in hemodialysis, requiring proper differential diagnosis of reactivation or acute infection, monitoring, and management of risk of reactivation due to persisting infection.

## References

[1] Makvandi M (2016). Update on occult hepatitis B virus infection. World J Gastroenterol.

[2] Fontenele AMM, Filho NS, Ferreira ASP (2013). Occult hepatitis B in patients on hemodialysis: a review. Ann Hepatol.

[3] Ramezani A, Aghasadeghi MR, Ahmadi F (2015). Isolated anti-hbc and occult HBV infection in dialysis patients. Nephrourol Mon.

[4] Raimondo G, Allain JP, Brunetto MR (2008). Statements from the Taormina expert meeting on occult hepatitis B virus infection. J Hepatol.

[5] Cuadrado GB, Calvo AA, Vibora EJ (2025). Position on the management of occult infection by hepatitis B virus (OBI) and anti-HBc + in hemodialysis units. Nefrologia (Engl Ed).

[6] European Association for the Study of the Liver (2025). EASL Clinical Practice Guidelines on the management of hepatitis B virus infection. J Hepatol.

[7] Tang Y, Liu X, Lu X (2020). Occult hepatitis B virus infection in maintenance hemodialysis patients: prevalence and mutations in "a" determinant. Int J Med Sci.

[8] Raimondo G, Locarnini S, Pollicino T (2019). Update of the statements on biology and clinical impact of occult hepatitis B virus infection. J Hepatol.

[9] Yip TC, Wong GL (2019). Current knowledge of occult hepatitis B infection and clinical implications. Semin Liver Dis.

[10] Albuquerque AC, Coelho MR, Lemos MF, Moreira RC (2012). Occult hepatitis B virus infection in hemodialysis patients in Recife, State of Pernambuco, Brazil. Rev Soc Bras Med Trop.

[11] Dzudzor B, Nsowah KK, Agyemang S (2024). Overt and occult hepatitis B virus infection detected among chronic kidney disease patients on haemodialysis at a Tertiary Hospital in Ghana. PLoS One.

